# PTSD and depression construct: prevalence and predictors of co-occurrence in a South Lebanese civilian sample

**DOI:** 10.3402/ejpt.v7.31509

**Published:** 2016-07-12

**Authors:** Laila F. Farhood, Souha Fares, Rachel Sabbagh, Carmen Hamady

**Affiliations:** 1 Hariri School of Nursing and Psychiatry Department, American University of Beirut, Beirut, Lebanon; 2 Hariri School of Nursing, American University of Beirut, Beirut, Lebanon

**Keywords:** posttraumatic stress disorder, depression, co-occurrence, war

## Abstract

**Background:**

Armed conflict, occupation, and political and economic instability that are particularly experienced by the civilian Lebanese population of South Lebanon would almost inevitably affect these individuals psychologically. Therefore, identifying predictors of co-occurring mental disorders is paramount to sound assessment and intervention planning.

**Objective:**

This study aims to determine the prevalence and predictors of co-occurring posttraumatic stress disorder (PTSD) and major depressive disorder (MDD) in a post-war population from South Lebanon.

**Method:**

A total of 991 citizens from 10 villages were interviewed using a cross-sectional design through random sampling. The prevalence of PTSD, MDD, or both was 23.4%. To identify predictors of PTSD and depression co-occurrence, multinomial logistic regression was used. Participants were divided into four groups (participants with no PTSD or depression, participants with PTSD only, participants with depression only, and participants with PTSD–depression comorbidity).

**Results:**

Among the significant predictors of PTSD–depression co-occurrence, female gender, health problems, social life events, and witnessed traumatic events were most consistently found. Additionally, employment and educational status, as well as social support, were found to significantly predict co-occurrence.

**Conclusions:**

Results reveal the distinct risk and protective factors that characterize the PTSD-depression profile. These findings will hopefully assist in the development of interventions that are sensitive to individuals’ psychosocial milieu.

**Highlights of the article:**

- Identifying predictors of PTSD-depression co-occurrence is paramount to sound assessment and intervention planning.
- Comorbidity was most strongly predicted by female gender, health problems, social life events, and HTQ witnessed events.
- Unemployment, having below secondary education, and low social support were also found to predict comorbidity.
- The substantial overlap in the risk factor profiles that were observed may suggest that PTSD and MDD co-occurrence represent a single general construct derived from traumatic stress.

Lebanon has repeatedly been shaken by multifaceted armed conflicts over the last few decades, leaving its population scathed at both the physical and psychological levels. Unfortunately, the chronology of several events has rendered South Lebanon a primary target for retaliatory attacks, resulting in thousands of casualties and injuries, while detrimentally influencing the mental health of its civilian survivors. In July 2006, South Lebanon faced a 33-day war. In an effort to end hostilities, the United Nations Security Council approved the UN resolution 1701 that was accepted by both parties. Consequently, individuals from South Lebanon exposed to the “July war” were vulnerable to several psychiatric disorders including posttraumatic stress disorder (PTSD) and depression (Farhood & Dimassi, 2010). Throughout this article, the terms “major depressive disorder (MDD)” and “depression” will be used interchangeably depending on what has been used in the literature.

PTSD has mostly been studied as a single disorder. However, individuals diagnosed with PTSD commonly reveal comorbidities with other psychiatric illnesses such as anxiety, alcoholism, and depression (Breslau, 2002; Heffernan, Andersen, Davidson, & Kinner, 2015). In the Australian national survey of mental health and well-being, individuals with PTSD were 5.2 times more likely to have an alcohol use disorder (AUD) compared to the rest of the population (Creamer, Burgess, & McFarlane, 2001; Mills, Teesson, Ross, & Peters, 2006). Similarly, over one-third (38%) of participants with MDD and AUD fit the criteria for PTSD (Bailey, Webster, Baker, & Kavanagh, 2012). These findings were consistent with another study conducted on the Rwanda Defense Forces (RDF) in Sudan, whereby the odds of PTSD co-occurring with MDD increased remarkably when there was a suspicion of high and harmful alcohol intake (Harbertson et al., 2013).

The comorbidity of PTSD with MDD could be considered a norm rather than an exception (O'Donnell, Creamer, & Pattison, 2014). In a systematic review of mental health among populations living in conflict in Libya, 50% of individuals with PTSD had severe depression (Charlson et al., 2012). Similarly, a meta-analysis of 57 studies (*n=*6,670) found that over half (*n=*3,391) of the participants exhibited PTSD–MDD comorbidity (Rytwinski, Scur, Feeny, & Youngstrom, 2013). Patients who exhibited this comorbidity often showed exacerbated symptoms, thereby corroborating the increasingly complex necessitation for treatment (Campbell et al., 2007). This highlights the need for studying the determinants of co-occurrence after exposure to traumatic events in order to identify threshold co-occurrence and ultimately seek more holistic treatments and preventive strategies.

Thus, to understand the increased prevalence of PTSD–MDD comorbidity, the first step would be to dissect the predictors of this co-occurrence. Identifying these factors would yield more comprehensive pharmacologic and psychotherapeutic treatment regimens. Farhood, Dimassi, and Strauss (2013) identified 11 factors associated with PTSD (age, gender, education, marital status, village, social support, financial problems, Harvard Trauma Questionnaire [HTQ] experienced, HTQ witnessed, cigarette smoking, and physical health problems) and seven with depression (village, employment, social support, financial problems, HTQ experienced, tranquilizer use, and physical health problems). Risk factors associated with the co-occurrence of these two conditions, however, were not identified. Conversely, O'Donnell et al. (2014) showed that a combination of risk factors could predict the prevalence of PTSD and MDD co-occurrence compared to PTSD alone. Specifically, these variables include event severity and individual characteristics such as psychiatric history, prior alcohol and substance use, and anxiety about the potential impact of the injury. Nevertheless, the current literature on risk factors for the co-occurrence of PTSD with MDD is still rudimentary, and no distinct profile for this co-occurrence has yet been identified.

## Objective

As previously described, most of the studies performed on psychiatric disorders in Lebanon have assessed the prevalence of PTSD or depression in civilian populations exposed to traumatic events (Farhood, & Dimassi, 2012; Farhood, Dimassi, & Lehtinen, 2006; Farhood, et al., 2013; Karam et al., 2014). These studies have not explicitly addressed the associated comorbidities and predictors for the co-occurrence of PTSD and depression. Using a secondary analysis of data collected by Farhood et al. (2013) from a sample of civilians from South Lebanon one year post-July 2006 war, the aim of this study is to determine risk as well as protective factors associated with co-occurring PTSD and depression.

## Method

### Study overview

The study uses a cross-sectional survey design and is part of a larger study, which targeted a sample of civilians from South Lebanon (Farhood et al., 2013). The primary objective of the original study was to examine the impact of war-related life events on the psychological well-being of a civilian population one year post-war. Inclusion criteria for participation involved those who were aged 20 or older and who were permanent residents of the selected areas for at least two years. The exclusion criterion involved any individual with a condition that may stand in the way of being interviewed, such as inability to communicate; yet none of the participants in this study met this criterion and thus nobody was excluded. The study employed random sampling with quotas to reflect the population distribution in the selected areas with regard to age and gender. Quotas were determined using the 2005 United Nations report (United Nations Interim Force in Lebanon, 2005). Trained interviewers conducted face-to-face interviews. Ethical approval for this study was granted by the American University of Beirut Institutional Review Board.

### Participants

This study included 991 subjects from 10 villages in South Lebanon. The sample size was calculated based on a previously used strategy (Farhood & Dimassi, 2012). The sample was diverse in age, gender, and education level. Furthermore, given the melting pot of religions that is found in Lebanon, a representative sample of diverse religions was considered for this sample.

### Measures

The measures used in this study were as follows.

#### Demographic and lifestyle variables

Demographic characteristics included gender, age, marital status, employment status, and education level. In addition, respondents were asked about their financial status, smoking (cigarette and argileh) habits, alcohol consumption, tranquilizer use, health problems (diabetes mellitus, triglycerides, digestive problems, hypertension, kidney disease, liver, cardiac disease, lung disease, cancer, or other) if any, and whether they had received psychiatric treatment in the past year.

#### Trauma exposure and PTSD

The Arabic version of the HTQ was used to assess exposure to traumatic events and PTSD threshold, rather than clinical diagnosis. The respondent was asked about 16 PTSD symptoms and was classified as having PTSD threshold if the 2.5 cutoff point was exceeded. This cutoff was initially recommended by Mollica et al. (1992) and later implemented extensively in other studies (Keller et al., 2003; Lhewa, Banu, Rosenfeld, & Keller, 2007), as well as in studies conducted by our research team in the south of Lebanon (Farhood, 2014; Farhood, & Dimassi, 2012; Farhood et al., 2006; Farhood, et al., 2013; Farhood, Dimassi, & Strauss, 2013). The HTQ has an inter-rater reliability of *r=*0.93 for traumatic events and *r=*0.98 for trauma symptoms; it has one-week test–retest reliability of *r=*0.89, *p<*0.0001 for traumatic events and *r=*0.92, *p<*0.0001 for trauma symptoms (Mollica et al., 1992). The sensitivity and specificity of the HTQ using a cutoff score of 2.5 were 78 and 65% respectively (Mollica et al., 1992). The Cronbach's alpha for the symptom part of the Arabic version, as calculated in previous analyses of the data (Farhood et al., 2013), was 0.87. The extent of exposure was quantified based on the HTQ manual, such that a total “exposure” score was computed for each participant. This version of the HTQ has already been used in Arabic-speaking populations and validated in other conflict zones (Farhood & Dimassi, 2012).

#### Depression

The Beck Depression Inventory (BDI) consists of 21 items that measure characteristics, attitudes, and symptoms of depression (Beck, Ward, Mendelson, Mock, & Erbaugh, 1961). Participants scoring above the cutoff point of 19 were classified as having moderate-to-severe depression. This cutoff score yielded a sensitivity and specificity of 87 and 88%, respectively, in a nationwide sample of healthy adults (Koivumaa-Honkanen, Kaprio, Honkanen, Viinamäki, & Koskenvuo, 2004). Similar to the HTQ, it is important to note that only threshold for depression can be derived from the BDI, not its diagnosis. The BDI has internal consistency ranging from 0.73 to 0.92 with a mean of 0.86. It has a split-half reliability co-efficient of 0.93, as well as high internal consistency with alpha coefficients of 0.86 and 0.81 for psychiatric and non-psychiatric populations, respectively (Beck, Steer, & Garbin, 1988). The BDI has been extensively used in Lebanese populations (Farhood et al., 2006; Farhood et al., 2013; Farhood & Dimassi, 2012).

#### Social support, life events, domestic violence, and displacement

Social support was assessed with eight items that asked participants about availability and improvement in social support after the 2006 war. The answers were coded 1 and 0, and summed to yield a social support score ranging from 0 (absence of social support) to 8 (strong social support). Life events were assessed with regard to social and financial problems that stemmed from the war. Social problems were measured using four items that asked participants about interpersonal problems at the workplace or interpersonal problems with friends or family. Financial problems were also assessed with four items: job loss, searching for a job, major change in income, and payment obligations or debt. Life events were individually scored from 0 to 4. Participants were asked whether or not they were displaced during the war and for how long. Moreover, they were asked if they had been exposed to domestic violence in the following areas: financial, social, physical, or psychological. Responses for these items were coded as 0 and 1; a new dichotomous variable “domestic violence” was later created with the value 0 representing no domestic violence and 1 indicating exposure to at least one type of domestic violence.

### Data analysis

Data analyses were conducted using Stata version 13 and SPSS version 21.0 for Windows. The dependent variable was classified into four categories: “none” (participants who had neither PTSD nor depression), “depression only” (participants who had depression only), “PTSD only” (participants who had PTSD only), and “co-occurrence” (participants with both PTSD and depression). Independent variables included the socio-demographic and lifestyle variables, trauma exposure, social support, domestic violence, displacement, and life events.

Characteristics of the study sample across the four diagnostic categories were summarized using descriptive statistics and compared using ANOVA, chi-square, and Fisher's exact tests as appropriate. The prevalence of co-occurrence was computed, as well as the prevalence of PTSD among individuals with depression and the prevalence of depression among individuals with PTSD.

Multinomial logistic regression was conducted to assess the unadjusted and adjusted associations between the dependent variable and the set of independent variables. Variables that were significant at the bivariate level were included in the stepwise multivariable regression model. Odds ratios and their 95% confidence intervals were reported. Significance level was set at α=0.05.

## Results

### Co-occurrence of PTSD and depression

Of the 991 subjects, 233 (23.4%) met the study criteria for PTSD and/or depression. Among those, co-occurrence of both PTSD and depression was found in 89 participants, which represent 9% of the total sample (Table 1). The prevalence of co-occurring PTSD and depression among individuals with PTSD was 51%; among individuals with depression, the prevalence of co-occurrence was 61%. Eighty-seven (8.8%) participants met the criteria for PTSD only (without depression) and 57 (5.6%) met the criteria for depression only (without PTSD). The number of participants who had co-occurrence was higher than those who had PTSD or depression alone. Characteristics of the participants across the four groups are shown in Tables 1 and 2.

**Table 1:** Frequencies and percentages of sample characteristics by diagnostic outcome

	None, *N*	PTSD alone, *N*	Depression alone, *N*	PTSD and depression, *N*	*p*
Sample size	758 (76.5%)	87 (8.8%)	57 (5.6%)	89 (9%)	
Gender					
Male	390 (51.5%)	23 (26.44%)	30 (52.6%)	26 (29.2%)	<0.001
Female	368 (48.5%)	64 (73.6%)	27 (47.4%)	63 (70.8%)	
Age					
20–29	261 (34.4%)	16 (18.4%)	13 (22.8%)	15 (16.9%)	<0.001
30–49	317 (41.8%)	53 (60.9%)	20 (35.0%)	41 (46.1%)	
50+	180 (23.8%)	18 (20.6%)	24 (42.1%)	33 (37.1%)	
Marital status					
Single	271 (35.8%)	29 (33.3%)	13 (22.8%)	20 (22.5%)	0.002
Married, other	487 (64.3%)	58 (66.7%)	44 (77.2%)	69 (77.5%)	
Employment					
Unemployed, housewife, other	391 (51.6%)	44 (50.5%)	38 (66.6%)	65 (73.04%)	<0.001
Employed	367 (48.4%)	43 (49.4%)	19 (33.3%)	24 (26.97%)	
Education					
Level 1	102 (13.5%)	10 (11.5%)	19 (33.3%)	21 (23.6%)	<0.001
Level 2	340 (44.9%)	45 (51.7%)	28 (49.1%)	48 (53.9%)	
Level 3	316 (41.7%)	32 (36.8%)	10 (17.5%)	20 (22.5%)	
Sufficient income for basic needs					
Most/all needs met	458 (60.4%)	50 (57.4%)	29 (50.9%)	30 (33.7%)	<0.001
Needs not met	300 (39.6%)	37 (42.5%)	28 (49.1%)	59 (66.3%)	
Cigarette					
Yes	213 (28.1%)	37 (42.5%)	22 (38.6%)	39 (43.8%)	0.001
Nargileh					
Yes	146 (19.3%)	12 (13.8%)	6 (10.5%)	13 (14.6%)	0.193
Alcohol					
Yes	105 (13.9%)	12 (13.8%)	3 (5.3%)	5 (5.6%)	0.048
Tranquilizer					
Yes	50 (6.6%)	7 (8%)	8 (14%)	31 (34.8%)	<0.001
Physical activity					
Yes	212 (28%)	24 (27.5%)	11 (19.3%)	14 (15.7%)	0.237
Domestic violence					
Yes	49 (6.5%)	15 (17.2%)	9 (15.8%)	18 (20.2%)	<0.001
Health problems					
Yes	219 (28.9%)	33 (37.9%)	28 (49.1%)	52 (58.4%)	<0.001
Displacement					
Yes	529 (69.8%)	66 (75.9%)	45 (78.9%)	65 (73%)	0.325

*Note*. Level 1: below primary education; Level 2: not completed secondary education; Level 3: secondary education or more competed.

**Table 2:** Means and standard deviations of sample characteristics by diagnostic outcome

	None, Mean (SD)	PTSD only, Mean (SD)	Depression only, Mean (SD)	PTSD and depression, Mean (SD)	*p*
Mean social support	6.42 (1.45)	5.94 (1.42)	5.68 (1.77)	5.44 (1.74)	<0.001
Social life events	0.25 (0.58)	0.47 (0.78)	0.53 (0.91)	0.71 (0.92)	<0.001
Financial life events	1.65 (0.97)	1.76 (0.98)	1.74 (1.03)	1.93 (0.93)	0.055
HTQ experienced	4.17 (2.32)	5.48 (2.42)	4.93 (2.68)	5.66 (2.5)	<0.001
HTQ witnessed	4.87 (2.45)	6.44 (2.7)	5.21 (2.23)	6.6 (2.8)	<0.001
HTQ heard	8.34 (3.32)	9.97 (3.96)	7.54 (3.6)	9.36 (3.5)	<0.001

### Unadjusted associations

#### Comorbid versus none

Participants with PTSD–depression comorbidity compared to participants with neither PTSD nor depression were more likely to be females (OR=2.57, 95% CI: 1.59–4.14), from older age groups including those who were aged 30–49 years (OR=2.25, 95% CI: 1.22–4.15) and those aged 50 and above (OR=3.19, 95% CI: 1.68–6.06), and married, divorced, or widowed (OR=1.92, 95% CI: 1.14–3.23). Those who had a below primary education (OR=3.25, 95% CI: 1.69–6.25) and those who did not complete secondary education (OR=2.23, 95% CI: 1.29–3.85) were more likely to have co-occurrence compared to individuals who completed secondary education or more. Similarly, cigarette (OR=2.00, 95% CI: 1.28–3.13) and tranquilizer (OR=7.70, 95% CI: 4.55–12.82) users were more likely to have a comorbid condition. Individuals whose needs were not met (OR=3.00, 95% CI: 1.53–4.76) were more likely to have comorbidity. Moreover, being exposed to at least one form of domestic violence, as well as having health, financial, and social life problems increased the risk of co-occurrence (OR=3.68, 95% CI: 2.04–6.67; OR=3.46, 95% CI: 2.21–5.43; OR=1.36, 95% CI: 1.08–1.70; and OR=2.19, 95% CI: 1.69–2.83, respectively). Finally, participants with a comorbid condition were more likely to be exposed to traumatic events: HTQ witnessed, HTQ heard, and HTQ experienced (OR=1.30, 95% CI: 1.19–1.43; OR=1.09, 95% CI=1.02–1.16; and OR=1.30, 95% CI=1.18–1.40, respectively). Conversely, compared to those who did not work, those who worked and those who reported more social support were at a lower risk of having a comorbid condition (OR=0.39, 95% CI: 0.24–0.64; and OR=0.69, 95% CI: 0.60–0.78, respectively).

#### Comorbid versus PTSD only

PTSD–depression comorbidity was distinguished from PTSD only by education (OR=3.36, 95% CI: 1.32–8.55), employment (OR=0.38, 95% CI: 0.20–0.71), tranquilizer use (OR=6.25, 95% CI: 2.52–14.93), health problems (OR=2.30, 95% CI: 1.26–4.22), and sufficient income (OR=2.66, 95% CI: 1.44–4.90). However, there was no significant difference between males and females who met the PTSD criteria in having a comorbid condition.

#### Comorbid versus depression only

Among those who met the criteria for depression, females (OR=2.69, 95% CI: 1.35–5.38), tranquilizer use (OR=3.27, 95% CI: 1.38–7.79), sufficient income (OR=2.04, 95% CI: 1.03–4.02), and HTQ witnessed (OR=1.23, 95% CI: 1.08–1.40) and heard (OR=1.17, 95% CI: 1.06–1.30) were significantly associated with co-occurrence.

### Stepwise multinomial logistic regression

Significant variables at the univariate level were entered into a multivariable forward stepwise multinomial logistic regression model. Although tranquilizer use and domestic violence were significant at the univariate level, they were excluded from the multivariate regression due to the small number of participants in each group, as this would affect model convergence. In a forward selection procedure, variables were entered in the model one at a time; at every new entry, all variables in the model were checked to see if any of them should be removed. This process was repeated until all remaining variables were significant at *p<*0.1 (Table 3).

**Table 3:** Stepwise multinomial logistic regression

	Adjusted odds ratio (95% CI)		
	
	None vs. comorbid	PTSD only vs. comorbid	Depression only vs. comorbid
Gender			
Male	1		1
Female	2.51 (1.35, 4.66)**		3.70 (1.60, 8.70)**
Employment			
Don't work	–	1	–
Work		0.29 (0.14, 0.63)**	
Education			
Level 3	1	–	
Level 1	1.80 (0.84, 3.80)		
Level 2	2.23 (1.22, 4.08)*		
Health problem			
No	1	1	–
Yes	2.98 (1.80, 4.93)**	1.96 (1.04, 3.70)*	
Social support score	0.67 (0.57, 0.76)**	–	–
Social life events	2.20 (1.64, 2.97)**	1.61 (1.17, 2.33)*	–
HTQ witnessed	1.28 (1.16, 1.41)**	–	1.22 (1.05, 1.42)**

*Note*. Level 1: below primary education. Level 2: not completed secondary education. Level 3: secondary education or more completed.

* *p*<0.05.

** *p*<0.01.

#### Comorbid versus none

Participants with PTSD–depression comorbidity differed significantly from participants with neither condition by gender; females were more than twice as likely to develop comorbidity compared to males (OR=2.51, 95% CI: 1.35–4.66). They also differed significantly on level of education; those who did not complete secondary education were more than twice as likely to be in the comorbid group compared to participants with secondary education or more (OR= 2.23, 95% CI: 1.22–4.08). In addition, having health problems, having experienced negative social life events (social and financial), and having witnessed trauma increased the risk of having a comorbid outcome (OR=2.98, 95% CI: 1.80–4.93; OR=2.20, 95% CI: 1.64–2.97; and OR=1.28, 95% CI: 1.16–1.41, respectively). On the other hand, participants who reported having more social support were protected from having a comorbid condition (OR=0.67, 95% CI: 0.57–0.76).

#### Comorbid versus PTSD only

Participants with PTSD–depression comorbidity differed significantly from those with PTSD only with regard to their employment status, health problems, and social life events. Specifically, all participants who were employed were protected from comorbidity compared to unemployed participants (OR=0.29, 95% CI: 0.14–0.63). Moreover, those who reported health problems were twice as likely to have comorbidity (OR=1.96, 95% CI: 1.04–3.70). Finally, those who reported more social life events were also twice as likely to exhibit comorbidity (OR=1.61, 95% CI: 1.17–2.33).

#### Comorbid versus depression only

As for participants who already had depression, females were almost four times as likely to have a comorbid condition compared to males (OR=3.70, 95% CI: 1.60–8.70). Participants who witnessed trauma were also significantly more likely to be in the comorbid group (OR=1.22, 95% CI: 1.05–1.42).

## Discussion

After the long-lasting retaliatory attacks that Lebanon has endured, havoc inevitably spread in almost every way possible, thereby detrimentally affecting its civilian survivors psychologically. In vulnerable populations that are frequently exposed to man-made disasters, it is crucial to investigate the influences of such experiences in order to discern a set of priority needs that can aid international agencies and local organizations in finding the best possible rehabilitation strategy. Therefore, in this study, the prevalence and associated predictors for comorbidity of PTSD and depression are addressed.

The relatively high prevalence of co-occurring PTSD and MDD that was found in the current study is consistent with other findings. Specifically, in a study conducted on male RDF military personnel, the prevalence of those who screened positive for both PTSD and depression and the prevalence of those who screened positive for PTSD only were almost equal (Harbertson et al., 2013). However, in contrast to previous studies that examined the prevalence and predictors of PTSD or depression in countries that had experienced war atrocities (Başoğlu et al., 2005; Canetti et al., 2010; de Jong et al., 2001; Hall et al., 2008; Priebe et al., 2010), in addition to studies similarly conducted on civilian populations in the south of Lebanon (Farhood, 2014; Farhood et al., 2006, 2013; Farhood & Dimassi, 2012; Farhood & Noureddine, 2003), we examined the predictors of the co-occurrence of PTSD and depression. Consistent with other findings (Ikin, Creamer, Sim, & McKenzie, 2010; O'Donnell et al., 2014), we propose that the reason behind the similar predictor variables and shared vulnerability for PTSD and depression that were found stems from the single general traumatic stress construct that they represent.

We found that the prevalence of co-occurring PTSD and depression (9%) was higher than that of PTSD (8.8%) or depression (5.6%) alone. We also found that the prevalence of co-occurring PTSD and depression among individuals with PTSD was 51%, which is very similar to what Ikin et al. (2010) found whereby 52% of participants who had PTSD experienced co-occurring depression. They proposed that the concept of a single traumatic stress construct involves a severity continuum whereby comorbidity occurs with more severe exposure to trauma compared to PTSD alone and is subsequently associated with more detrimental mental health outcomes (Ikin et al., 2010). There is evidence that suggests that the development of MDD stems from preexisting vulnerabilities and subsequent psychosocial stressors, whereas the development of PTSD stems from stressors pertaining to disaster-related events (Javidi & Yadollahie, 2011; Miguel-Tobal et al., 2006; Tracy, Norris, & Galea, 2011). We similarly found that depression was not associated with trauma exposure; rather, it was associated with psychosocial factors such as unemployment, health problems, and social life events. Those findings are in line with Ikin et al.'s (2010) explanation of a severity continuum of this general traumatic construct. Most participants displayed co-occurrence possibly due to the culminating stressors that result from war traumas, in addition to not seeking psychological or psychiatric help over time, which could thereby exacerbate the problem and yield comorbidity.

The multinomial logistic regression revealed that female gender, health problems, social life events, and witnessed traumatic events were the most consistent predictors of co-occurrence. Nevertheless, we also found that co-occurrence was significantly predicted by low social support, low education level (below secondary education), and unemployment.

Female gender predicted comorbidity among those who had neither PTSD nor depression, as well as among those with depression only. These gender differences may be traced to the disruptions of daily functions, including curfews and food shortages that are usually faced in times of war, and which may be particularly felt by women. Similar to our findings whereby there was no significant gender difference between those with PTSD only and those with co-occurrence, Rytwinski et al. (2013) reported that nearly equal numbers of males and females diagnosed with preexisting PTSD were also found to have MDD, with both genders equally likely to develop co-occurrence. Our findings thereby corroborate the higher likelihood of females to develop PTSD or comorbidity after exposure to trauma compared to males (Elklit, Østergård, Lasgaard, & Palic, 2012; Galea, Tracy, Norris, & Coffey, 2008; Priebe et al., 2009; Tekin et al., 2016). Alternatively, this gender disparity could be explained by the greater tendency of women to express and report feelings of fear (Priebe et al., 2009); men, in contrast, might perceive disclosure as a threat to masculinity and consequently might choose to conceal their feelings.

Health problems in our study rendered individuals at two to three times the risk for developing comorbidity compared to those with PTSD only and those with neither PTSD nor depression, respectively. Studies have shown that physical pathology and psychopathology often coexist immediately following a traumatic event (Galea, Nandi, & Vlahov, 2005). The direction of causality between health problems and mental health outcomes, if any, is obscure, especially if any mediating variables are to be considered. Health problems could increase the risk of developing PTSD and depression; or, alternatively health problems could be exacerbated by symptoms of PTSD and depression. Furthermore, disadvantaged socioeconomic lifestyles may intervene in the physical–mental health association. It is possible that financial problems lead to more physical health problems, which in turn would lead to worse mental health outcomes (Matthews & Gallo, 2011). It is also possible that because those with financial problems were more likely to utilize health services, and those who utilized these services were more likely to have PTSD (Farhood & Noureddine, 2003), those with disadvantaged socioeconomic lifestyles are at increased risk of developing both physical and mental health conditions (World Health Organization [WHO], 2000).

The significant association between trauma witnessing and comorbidity is remarkable. Individuals in our study who had witnessed trauma without depression or PTSD, and those with depression only were approximately 1.5 times more likely to develop a comorbid condition. Those findings are in accordance with the assumption that determinants of co-occurrence are related to the level of exposure to torture, terror, and traumatic events (Farhood & Dimassi, 2012; Farhood et al., 2006, 2013). The association between trauma exposure and PTSD, as well as trauma exposure and PTSD–depression co-occurrence, may entail that the traumatic stress that ensues from these war-related events is associated with PTSD; however, when exacerbated by other antecedents, depression may arise, rendering the individual at greater disadvantage. The possibility that one disorder could increase susceptibility to the other is indicative of a shared vulnerability profile for both disorders, with comorbidity associated with both trauma witnessing and associated psychosocial stressors that often ensue.

Negative social life events and low levels of social support were found to be associated with co-occurrence. Strong supportive networks fortunately characterize the Arab world. Nevertheless, negative social life events may be reported, which in our study had doubled the individual's risk of meeting threshold for comorbidity. Social support, on the other hand, served as a protective factor. Interpersonal problems at the workplace and/or interpersonal problems with friends, children, or marital relations may act as a barrier to expressing one's concerns and to feel supported by those who are closest to the individual. Social networks and social support systems could thereby moderate the influence of stressful life events by enabling individuals to share their concerns, to feel secure, and to feel that they actually belong somewhere (Dressler, 1985; Goldberg, Van Natta, & Comstock, 1985). Thus, high levels of social support act as a protective factor for PTSD (Farhood & Noureddine, 2003; Weiss, Garvert, & Cloitre, 2015) and depression (Nillni et al., 2013). Although social support correlates with psychological resilience, its effectiveness is not ubiquitous, for it depends on the type of support provided, as well as the extent to which an individual's needs are met (Southwick et al., 2016). It is also crucial to note that the relationship between PTSD–depression comorbidity and social support does not highlight the direction of causality between these variables. It is possible that individuals with comorbidity are more likely to be socially withdrawn and thereby acknowledge and report less utilization of social support.

Level of education and unemployment were also significant predictors of comorbidity in our study. Individuals with below secondary education were twice as likely to develop comorbidity. Furthermore, those with PTSD who were unemployed were at significantly greater risk of developing comorbidity. It can be argued that unemployed individuals usually have no source of income; this financial burden, in turn, could predict depression among those with PTSD only. Furthermore, those with lower education may not have the cognitive capacity to cope with traumatic events (Priebe et al., 2009) and therefore may not adopt healthy coping strategies in such difficult times. The enduring cumulative stressors and negative life events would henceforth influence these individuals’ long-term coping and dealing with financial and social hardships (Farhood et al., 1993).

Although in the univariate regression we found that tranquilizer use could place the individual at eight times the risk of meeting threshold for comorbidity, we did not include this in our multivariable regression due to the small number of reports. We also did not include alcohol use in both the univariate and multivariate regressions for that reason. This low number may be attributed to the traditional culture that characterizes Lebanon, whereby alcohol use – and possibly also tranquilizer use – is negatively regarded and prohibited by some religious communities in the South Lebanon.

Domestic violence, although significant at the univariate level, was similarly excluded from the multinomial logistic regression due to the small number of subjects in this category. This low number might reflect the preference of Lebanese not to disclose personal issues to strangers and to not exaggerate their problems by making them actually appear as “problems.” Nevertheless, those who reported domestic violence were approximately four times at higher risk for developing comorbidity. This finding validates the literature that exposure to domestic violence may lead to PTSD and depression symptomology (Griffing et al., 2006; Peckover, 2002).

Nevertheless, one limitation in this study common to all research using self-reports is that recall bias may be involved. Participants may underreport predictors and events that relate to their condition due to embarrassment, shame, or social desirability. Cross-sectional methods may lead to common method biases, especially since in this study self-report measures were the sole type of data collected. Given the limitations associated with cross-sectional methods, future studies could alternatively follow the sample over a period of time to provide prospective evidence concerning the intricate relationship between demographic and lifestyle variables, trauma exposure, social support, displacement, and the risk of developing PTSD and MDD co-occurrence. Nonetheless, the use of a cross-sectional design in our study was adequate for the present purposes given that it is the most useful and fastest way to study prevalence and identify associations between variables (Mann, 2003).

Additionally, although screening questionnaires are often helpful, the cutoff scores selected may need further scrutiny (Turner, Bowie, Dunn, Shapo, & Yule, 2003). In a validation study conducted on a small sample of 51 former Vietnamese political prisoners, a cutoff score of 1.17 for the total scale of the HTQ yielded excellent diagnostic accuracy (sensitivity of 98% and specificity of 100%). Conversely, when the 2.50 cutoff was used, sensitivity and specificity were, respectively, found to be 16 and 100% (Smith et al., 1997). Therefore, it would be optimal for future community-based studies conducted among populations exposed to war traumas to include a validation sub-study. This, in turn, would allow researchers to discern the most adequate cutoff score for these specific populations prior to their implementation of the study (Smith et al., 1997).

Despite these limitations, to our knowledge this study is the first to demonstrate distinct risk factor profiles for PTSD–depression comorbidity, PTSD only, and depression only among residents in South Lebanon. Moreover, with a very large random sample of 991 adults from various backgrounds, the results from this study could be generalized to other war-torn communities. This study also used validated measures that were already administered in previous studies on Arabic-speaking populations. Results from this study indicate a clear need for future psychological interventions to be implemented in vulnerable populations exposed to war traumas and continuous threats, as well as a need to assess the implications of these interventions. In fact, our research team has recently implemented a mental health intervention through community-based educational workshops conducted with teachers and parents in the south of Lebanon, with the aim of ultimately preventing chronic symptoms and any long-term effects of traumatic events.

Additionally, given that PTSD and depressive symptoms generally impair daily functioning, the relatively high prevalence rates observed may entail that the working capacity and the functioning of these communities would subsequently decrease. Thus, their co-occurrence would not surprisingly yield even more detrimental outcomes. Public health officials and developmental planners for these concerned areas should thereby consider these socioeconomic implications, while being aware of a general traumatic stress construct continuum with different symptom severities and outcomes. It may also be interesting for future studies to investigate how the waning economic situation that is experienced during and post-war mediates the effect of traumatic events.

## Conclusions

To sum up, distinct profiles for comorbidity among subjects with neither PTSD nor depression, depression only, or PTSD only could be derived from these findings. When faced with war atrocities, comorbidity could most strongly be predicted by female gender, health problems, social life events, and HTQ witnessed events. Unemployment, having below secondary education, and low social support were also found to predict comorbidity. The substantial overlap in the risk factor profiles that were observed may suggest that PTSD and MDD co-occurrence represent a single general construct derived from traumatic stress, with some predictors common to both conditions, and a few specific to PTSD or MDD.

In conclusion, it is recommended that these findings be used for policy making and interventions. Mental health interventions that meet the basic mental health needs of its residents during times of conflict and peace are needed. Awareness campaigns that seek to enhance the education and health status of civilians and that also target some of the strongest predictors of threshold co-occurrence that we have observed, such as health problems, could be implemented. Fortunately, this culture treasures and values social support, which has been consistently shown to help individuals cope with the atrocities and traumas that stem from wars. Micro-policies that are sensitive to the socio-cultural milieu and that enhance the social support that is available to families should therefore be executed. Governmental and non-governmental agencies should aim at providing assistance for vulnerable families by assisting in their basic economic needs, as well as counseling services that aid in coping with the emanating war-related stressors that often persist in these families.
